# Beyond comfort: why breast support and protection belong in women's sport injury prevention

**DOI:** 10.3389/fspor.2026.1882792

**Published:** 2026-07-01

**Authors:** Jun Woo Kwon

**Affiliations:** Sports Technology Laboratory, Department of Physical Education, Seoul National University, Seoul, Republic of Korea

**Keywords:** breast injury surveillance, breast protection, breast support, sports bra fit, women's sport injury prevention

## 1 Introduction

Breast support and breast protection have usually been treated as matters of comfort, apparel fit, or participation in women's sport. This framing is too narrow if breast-related problems influence pain, movement, reporting behaviour, protective equipment use, and exposure to contact or impact.

In women's sport, injury-prevention discussions have often centred on established topics such as anterior cruciate ligament injury, relative energy deficiency in sport, menstrual health, training load, and bone stress injury. However, breast-related concerns have not been integrated into injury-prevention frameworks with the same consistency, despite being distinctly female-specific. Recent reviews indicate that breast injuries occur in women's sport, particularly in contact and collision contexts, while the literature remains limited and fragmented (1, 2). Broader work on female-specific health challenges in elite sport also suggests that breast concerns sit alongside other under-recognised issues affecting performance, wellbeing, and athlete support (3). Systematic evidence on breasts and bras in physical activity shows that breast-related factors are relevant to pain, participation, and movement-related outcomes, not merely clothing preference (4).

Breast-related concerns warrant consideration within women's sport injury-prevention and athlete-health research because they are documented, female-specific, potentially modifiable, and insufficiently captured in current systems. Existing evidence supports well-fitted sports bras for reducing breast movement and exercise-induced breast pain (5, 6), whereas contact breast injury prevention and protective-equipment efficacy require further sport-specific investigation. Treating breast support and protection only as comfort issues may obscure direct and indirect pathways through which breast pain, injury, support, protection, reporting, and equipment fit interact.

## 2 Breast-related concerns are not merely comfort issues

The term “comfort” can make breast-related problems sound minor. In sport, discomfort is rarely minor when it changes how an athlete trains, competes, reports symptoms, or chooses equipment. Breast pain has been reported by elite female athletes and associated with perceived performance limitations (5). Exercise-induced breast pain also appears related to physical characteristics and sports bra use, suggesting a problem shaped by fit, support, and sport demands (6). Similar concerns have been described beyond elite sport, with active females reporting breast pain as a relevant issue in physical activity contexts (7).

Breast injuries further challenge the idea that this is only a comfort issue. In contact football, breast injuries have been reported across football codes, playing positions, and competition levels, with athletes describing effects on participation and performance (8). Breast injuries have also been documented among collegiate athletes in basketball, soccer, softball, and volleyball, indicating that the issue is not confined to one sport or athlete group (9). Because these injuries may not always lead to time loss, breast bruising, impact-related pain, or training modification due to breast discomfort may remain less visible in conventional surveillance systems.

Breast-related concerns sit between pain, participation, performance, equipment, and injury. The issue is not whether every episode of breast pain should be classified as a sports injury, but whether injury-prevention systems can ignore female-specific, modifiable, and underreported problems that may shape exposure and behaviour. Future research should therefore avoid treating these factors as peripheral solely because previous studies have often framed them through comfort, fit, or participation.

## 3 Plausible pathways linking breast support and protection to injury prevention

**Figure 1:**
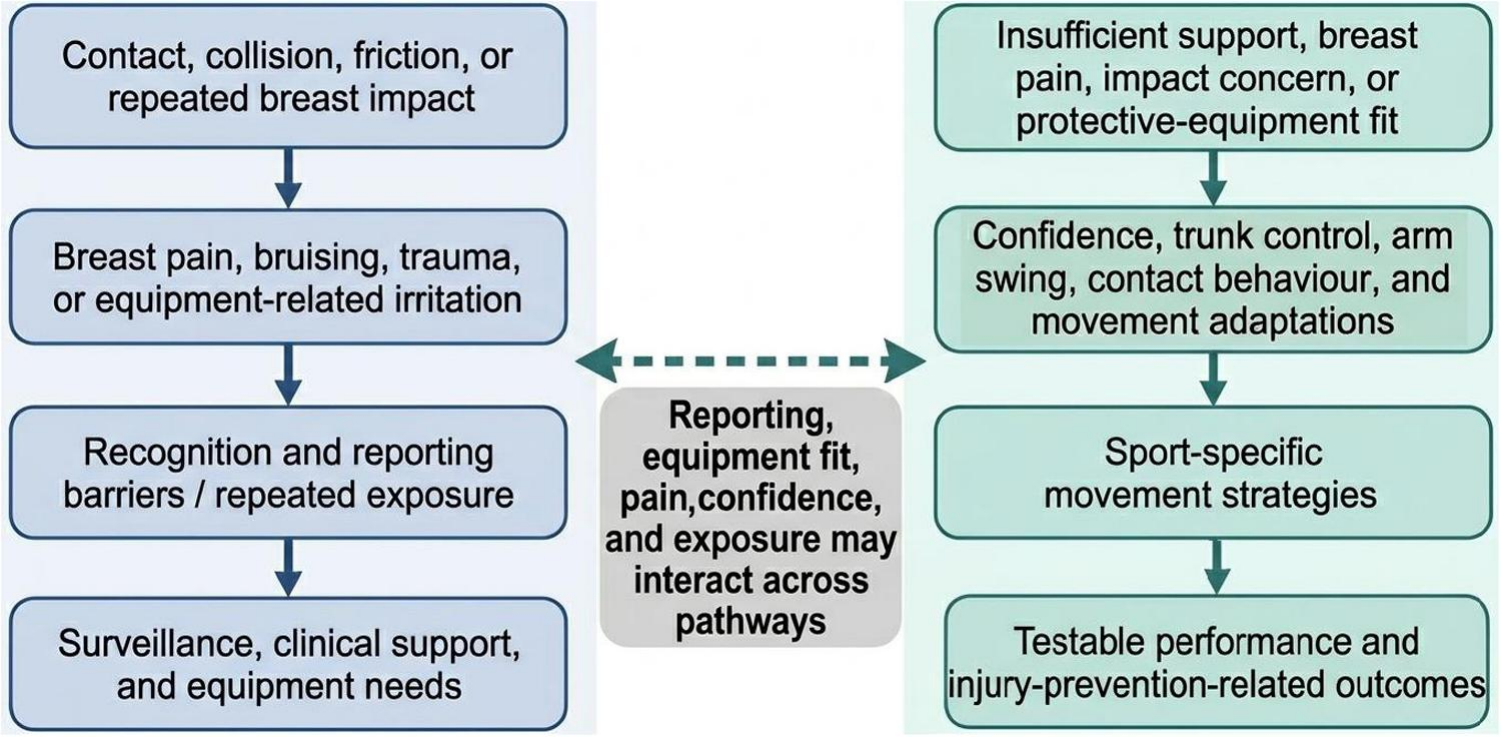
Proposed interacting pathways through which breast support, protection, reporting, and equipment fit may inform women's sport injury-prevention research. Note. The pathways are presented for conceptual clarity and are not proposed as independent causal mechanisms. Reporting, equipment fit, pain, confidence, and exposure may interact across pathways. The figure identifies variables that should be made visible, measurable, and testable rather than established injury-prevention effects.

Breast support and protection can be brought into injury-prevention research through two plausible pathways: a direct breast injury pathway and an indirect biomechanical-behavioural pathway (Figure 1).

In contact, collision, and ball sports, the breast may be exposed to impact, compression, or repeated contact, while friction-related breast or bra irritation can occur across sports through skin, garment, or equipment interaction (8, 9). These exposures may be associated with breast pain, bruising, or other breast injuries. Evidence from contact football and broader reviews of breast injury in women's sport suggests that this pathway warrants further attention, even if surveillance remains incomplete (1, 2, 8). The point is not that every breast impact produces a clinically significant injury. It is that breast impact is a sport-related exposure rarely given the same injury-prevention attention as other body regions.

The indirect pathway is less proximal to breast injury itself, but easily overlooked. Breast support may influence how athletes move. Experimental work has shown that greater breast support can alter trunk and knee joint biomechanics during landing tasks commonly discussed in relation to anterior cruciate ligament injury risk (10). Related work has linked increased breast support with altered knee joint stiffness and knee biomechanics during treadmill running (11), while other studies show that breast support affects breast kinematics across running and exercise modalities (12, 13). These findings do not show that breast support prevents lower-limb injury, but they support the rationale for measuring breast-related factors in studies of movement patterns relevant to injury prevention.

This indirect pathway should also include behaviour. If athletes experience breast pain, worry about contact, or feel inadequately supported, they may alter arm swing, trunk position, running mechanics, jumping confidence, or contact behaviour. Such adaptations may be subtle, sport-specific, and sometimes irrelevant to injury. Breast support has even been associated with running economy outcomes, suggesting effects beyond perceived comfort into movement and performance-related domains (14). For injury-prevention research, the question is how, when, in whom, and in which sport contexts breast pain, breast support, bra fit, impact concern, and protective-equipment fit influence movement, confidence, contact behaviour, participation, and injury-related outcomes.

These pathways should be distinguished, but not treated as independent or mutually exclusive. The direct pathway concerns breast pain or injury arising from impact, compression, friction, or repeated exposure. The indirect pathway concerns how breast-related pain, insufficient support, concern about impact, or protective-equipment fit may influence confidence, trunk control, arm motion, contact behaviour, or sport-specific movement strategies. Importantly, equipment intended to reduce direct contact exposure may also alter movement or behaviour. The purpose of separating the pathways is conceptual clarity, not a claim that they can always be evaluated in isolation.

## 4 Making breast support and protection visible in research and practice

If breast support and protection are to be taken seriously in injury-prevention research and practice, they first need to be made visible through routine questions and recording categories. Surveillance can improve visibility by documenting breast pain, bruising, contact breast injury, impact-related discomfort, equipment-related irritation, bra-fit problems, protective-equipment use, and training modification due to breast discomfort. Current injury definitions and reporting systems can miss problems that do not cause time loss, even when they affect training, confidence, or exposure (15, 16). These breast-related variables may not always meet narrow injury definitions, but they can identify modifiable support, protection, clinical, and education needs. This is particularly important for female athlete health domains because breast pain and injury, like menstrual or pelvic-floor symptoms, may affect performance or require training modification without causing absence (17, 18). Breast-related problems should therefore be considered within non-time-loss, medical-attention, symptom-based, and performance-related surveillance approaches (15–18). Coding is another source of invisibility: breast-related problems may disappear when grouped within broad chest categories or when breast-specific diagnostic codes are absent. Updated sports medicine coding systems provide the infrastructure for comparable injury and illness recording (19). More recent OSIICS version 16 updates added breast-specific codes within the chest region and recommend separating general chest injuries from breast-specific CXB codes when reporting injuries (20). This is important for estimating breast injury rates and informing equipment decisions and sport-specific protection regulations.

Visibility also depends on whether athletes recognise breast pain or injury as reportable and whether they feel able to disclose it. Breast-related problems may be minimised, treated as part of the game, or kept private rather than discussed clinically (21, 22). The aim should be to normalise breast-health conversations and reporting routes, not to normalise breast pain or injury as inevitable (22, 23). In under-18 international female rugby players, contact breast injury and exercise-induced breast pain were common, yet most contact breast injuries were not reported and none were reported to coaching or medical personnel (21). Qualitative work with women's rugby stakeholders further suggests that reporting may be constrained by low awareness, topic sensitivity, lack of clear reporting pathways, limited access to female support staff, and androcentric sport cultures in which female-specific health issues are marginalised (22). Education-intervention evidence also indicates that targeted breast-health education can improve knowledge, comfort, and intended reporting behaviour among players and support staff (23). Athlete health screening and education should therefore create safe, routine, and age-appropriate routes for discussing breast pain, breast injury, bra fit, and protective-equipment use.

A second priority is equipment, but this must be discussed within sport-specific regulatory contexts. Breast protective equipment is not uniformly permitted, validated, or mandated across sports; in some sports, competition use may depend on governing-body approval, whereas in others, breast protection may be compulsory. Differentiating breast injuries from broader chest injuries is also important for evidence-informed equipment decisions and sport-specific protection regulations (20). Studies of female contact football players show that breast protective equipment is not consistently used, and that fit, comfort, awareness, and perceived need may influence use (24). Breast and torso characteristics vary across athletes, with implications for sports bra and breast-protection design (25). Evidence from adjacent protective-equipment contexts, such as body armour research in female soldiers, also illustrates how torso and breast characteristics can shape perceived fit and protection (26). Breast protection should therefore not be treated as a generic add-on; it needs to be legal, fitted, sport-specific, acceptable to athletes, and evaluated under real movement and contact demands.

Education is another modifiable intervention point. Bra education has been shown to improve bra knowledge, fit, and support level in adolescent female athletes (27). More recent elite-sport interventions have also shown that breast and bra education or individual sports bra prescription can improve knowledge, fit, comfort, and perceived support among female athletes and match officials (28–30). Existing intervention evidence should not be understated: breast and bra education, individual assessment, and sports-bra prescription can improve knowledge, fit, support, comfort, bra-related issues, and, in some settings, breast pain (27–30). This evidence supports breast support as a modifiable athlete-health intervention, while contact breast injury prevention and protective-equipment efficacy remain separate questions requiring sport-specific testing (24, 25).

Future research should combine awareness, reporting, and testing rather than treating these as sequential or competing priorities. Awareness and education are prerequisites for valid surveillance and intervention research: if athletes, coaches, and support staff do not recognise breast pain or contact breast injury as relevant, these problems are unlikely to be reported or measured (22, 23). At the same time, awareness alone is insufficient. Following team-sport injury-prevention models, field studies should examine breast impact exposure, protective-equipment use, and reporting; laboratory studies should test whether support level alters landing, running, cutting, or contact-related mechanics; and intervention studies should evaluate whether bra fitting, breast-health education, or protective-equipment programmes improve pain, confidence, reporting, and movement-related outcomes (31). Wearable breast-impact monitoring may make some of this work more feasible in real sport settings (32). The immediate goal is not to claim injury reduction, but to make breast-related variables visible, measurable, and testable.

## 5 Discussion

Breast support and protection should not be presented as established injury-prevention interventions. The evidence is not yet strong enough for that claim. A more appropriate position is that breast pain, breast injury, bra fit, protective-equipment use, and reporting barriers are female-specific, potentially modifiable, and insufficiently captured in current injury-prevention research. Framing breast support and protection only as comfort or apparel preference may overlook a relevant area of athlete health.

Future studies should describe the prevalence and reporting of breast pain and breast injuries across sports; include non-time-loss and medical-attention problems in surveillance; evaluate the usability, legality, and protective value of breast protective equipment; and test whether education, fitting, and athlete-centred screening improve reporting, comfort, confidence, and movement-related outcomes. Moving breast support and protection beyond comfort is therefore not a claim of proven injury reduction, but a step toward more inclusive and female-specific injury-prevention questions in women's sport.
